# The Two-Wrongs model explains perception-action dissociations for illusions driven by distortions of the egocentric reference frame

**DOI:** 10.3389/fnhum.2015.00140

**Published:** 2015-03-18

**Authors:** Paul Dassonville, Scott A. Reed

**Affiliations:** Department of Psychology and Institute of Neuroscience, University of OregonEugene, OR, USA

**Keywords:** vision, illusion, perception, action, reference frame, saccade, motor control

## Abstract

Several studies have demonstrated a dissociation of the effects of illusion on perception and action, with perception generally reported to be susceptible to illusions, while actions are seemingly immune. These findings have been interpreted to support Milner and Goodale's Two Visual Systems model, which proposes the existence of separate visual processing streams for perception and action. However, an alternative interpretation suggests that this type of behavioral dissociation will occur for any illusion that is caused by a distortion of the observer's egocentric reference frame, without requiring the existence of separate perception and action systems that are differently affected by the illusion. In this scenario, movements aimed at illusory targets will be accurate if they are guided within the same distorted reference frame used for target encoding, since the error of motor guidance will cancel with the error of encoding (hence, for actions, two wrongs *do* make a right). We further test this Two-Wrongs model by examining two illusions for which the hypothesis makes very different predictions: the rod-and-frame illusion (which affects perception but not actions) and the simultaneous-tilt illusion (which affects perception and actions equally). We demonstrate that the rod-and-frame illusion is caused by a distortion of the observer's egocentric reference frame suitable for the cancellation of errors predicted by the Two-Wrongs model. In contrast, the simultaneous-tilt illusion is caused by local interactions between stimulus elements within an undistorted reference frame, precluding the cancellation of errors associated with the Two-Wrongs model such that the illusion is reflected in both perception and actions. These results provide evidence for a class of illusions that lead to dissociations of perception and action through distortions of the observer's spatial reference frame, rather than through the actions of functionally separate visual processing streams.

## Introduction

Although the visual processes of the brain ultimately create a unified percept of the visual world, there exists a clear modularity in the cortical systems that underlie vision. The flow of information through these modular structures appears to bifurcate and become two prominent cortical streams after leaving primary visual cortex, with one stream veering ventrally into the temporal cortices and another veering dorsally into the parietal cortices. These streams were originally thought to be responsible for the processing required to separately determine the identity of objects (the ventral *what* pathway) or their locations (the dorsal *where* pathway, Ungerleider and Mishkin, 1982). Later, though, this dissociation of function was reinterpreted by Milner and Goodale (1995), who suggested that both streams encode object- and space-based attributes of the visual world, but for different purposes. In their Two Visual Systems model, the ventral stream was thought to specialize in the perceptual and cognitive processes that underlie conscious perception and visual cognition, while the dorsal stream was thought to be dedicated to the guidance of goal-directed actions.

One type of finding that has been touted as evidence for the Two Visual Systems model is a dissociation of illusion susceptibility in the perceptual reports and actions of healthy individuals, with perception often susceptible to illusions for which actions are immune (or, at least, less affected). For example, Bridgeman et al. (1997) asked observers to make categorical judgments about the location of a visual target by pressing one of five response keys to indicate which of an array of possible locations the target occupied in a given trial. When the task was performed in the presence of a large rectangular frame whose center was offset from the observer's objective midline, the target was typically mislocalized in the direction opposite the frame shift, with, for example, a right-shifted frame causing the participants to report that the target occupied a position that was to the left of its actual location (the induced Roelofs effect, a variant of an illusion originally discovered by Roelofs, 1936). However, when these same observers were asked to make immediate, open-loop pointing movements to the target, these sensorimotor responses were accurate, suggesting that the sensorimotor systems are immune to the illusions to which perception is prone. Similar dissociations of illusion susceptibility have also been reported for, for example, the Ebbinghaus illusion (Aglioti et al., 1995) and the rod-and-frame illusion (Dyde and Milner, 2002), although some of these findings (or their interpretations) have been disputed (see Schenk and McIntosh, 2010, for a review). Thus, the Two Visual Systems model suggests that an isolation of perceptual and sensorimotor functions within separate cortical processing streams would allow for a dissociation of the effects of illusion (Milner and Goodale, 1995).

Although the Two Visual Systems model provides one possible means to account for the dissociation of the effects of illusions on perception and action, it is important to consider alternative explanations that may also account for these findings—an effort that is greatly aided by a full consideration of the visuospatial basis of the illusion in question. In the case of the induced Roelofs effect (Bridgeman et al., 1997), the perceptual illusion can be attributed to the offset frame's tendency to distort the observer's egocentric reference frame, by pulling the observer's apparent midline in the direction of the offset (Dassonville and Bala, 2004; Dassonville et al., 2004). The observer's assessment of the target's location within this distorted reference frame causes the target to appear to occupy a location that lies in a direction shifted opposite the frame. As a specific example, a frame offset to the observer's right causes a rightward bias in the apparent midline. In turn, a target presented directly in front of the observer would appear to lie to the left of straight ahead, when compared to the rightward-biased apparent midline.

If the perceptual effect of the induced Roelofs illusion is accounted for by a distortion of the observer's egocentric reference frame, what would be the expected effect of this distortion on actions guided toward the target? As demonstrated in our earlier study (Dassonville and Bala, 2004), guidance of the movement within the same distorted reference frame causes a second error that cancels the error of target encoding. As an example, imagine again a target presented directly in front of the observer, within a frame that is shifted to the observer's right such that the target is incorrectly perceived to lie some distance to the left of the distorted apparent midline. If, now, the observer makes an eye movement aimed at a location that same distance to the left of the distorted apparent midline, the error in oculomotor guidance will cancel with the earlier error of target encoding, resulting in an accurate movement (a similar cancellation of errors occurs if a pointing movement of the hand is guided within the same distorted reference frame, Dassonville et al., 2004). Thus, this cancellation of the errors of perceptual encoding and motor guidance causes a dissociation of the effects of the illusion—with accurate actions in spite of perceptual illusions—without requiring the existence of separate visual systems that are differently susceptible to the illusion, as is suggested by the Two Visual Systems model (Milner and Goodale, 1995). In the lab, we have begun to use the term “the Two-Wrongs model” to refer to this idea that when a sensorimotor response is guided within the same distorted reference frame as that used to encode the illusory location of a target, the resulting cancellation of errors would allow for accurate movements (in essence, “two wrongs *do* make a right,” to paraphrase the title of a related study by Li and Matin, 2005).

The cancellation of errors that would allow for an accurate sensorimotor response in spite of a perceptual illusion in the Two-Wrongs model would only occur if the movement is evoked while the distortion of the egocentric reference frame still exists. However, if the movement is significantly delayed after target encoding, circumstances could allow the illusion-induced distortion of the reference frame to subside before movement completion. This would cause a mismatch between the errors of target encoding and movement guidance, such that the cancellation of errors would be incomplete, and the illusion would then be expected to affect the accuracy of the sensorimotor response. Indeed, if a 4-s delay is mandated between the offset of a Roelofs-inducing frame (and its enclosed target) and the subsequent motor response, the initially-distorted apparent midline drifts back toward veridical under the influences of vestibular (Morant, 1959) and proprioceptive cues (Karnath, 1999), and, as a result, a memory-guided movement to the target is biased by the now-unmatched illusion of target encoding (Dassonville and Bala, 2004; Dassonville et al., 2004). This should not suggest that all memory-guided responses would reflect the illusion, however: if the target is flashed but the Roelofs-inducing frame remains present during the delay period, its continued presence maintains the egocentric reference frame in its biased state, allowing for the cancellation of errors and accurate responses predicted by the Two-Wrongs model, even after the delay (Dassonville and Bala, 2004).

**Figure 1:**
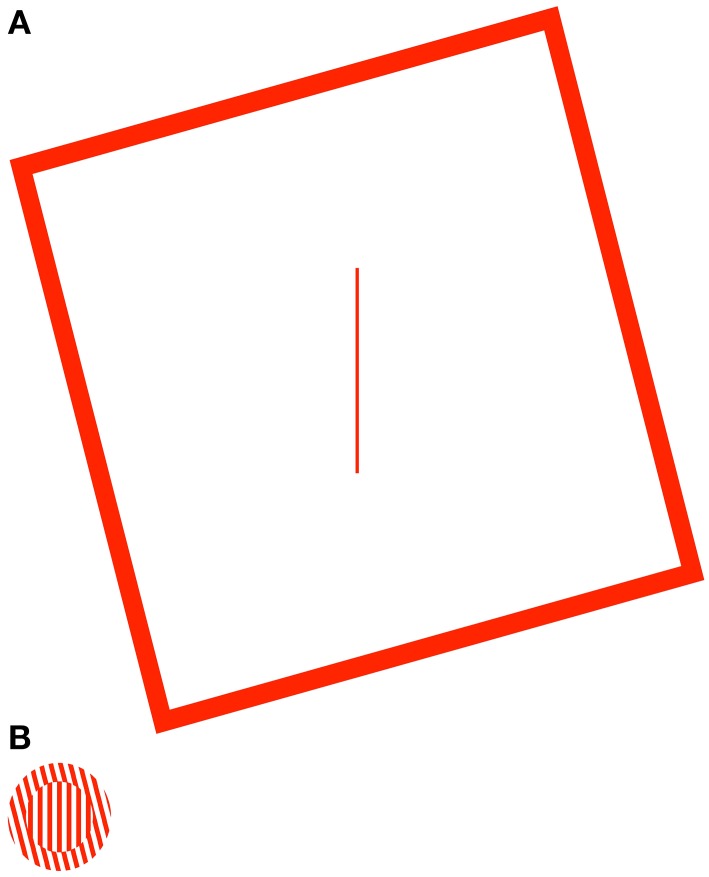
**The rod-and-frame (A) and simultaneous-tilt illusions (B)**. In the rod-and-frame illusion (RFI), the tilted frame typically causes the central rod (here vertical) to appear tilted in the opposite direction. In the simultaneous-tilt illusion (STI), the tilted surround grating typically causes the central grating (also vertical) to appear tilted in the opposite direction.

The mechanisms of the Two-Wrongs model should generalize beyond the induced Roelofs effect, and hold true for any illusion that is specifically caused by a distortion of the observer's egocentric reference frame. Indeed, a similar mechanism has been used to explain the dissociation in perception and action that is observed with illusions of target height (Li and Matin, 2005) and orientation (Li et al., 2008) caused by visible frames that are tilted in the pitch and roll axes, respectively. In the present study, we will more specifically test the assumptions of the Two-Wrongs model, by comparing the effects of two illusions of orientation, the rod-and-frame illusion (RFI; Witkin and Asch, 1948) and the simultaneous-tilt illusion (STI; Gibson, 1937). In the RFI, the clockwise or counterclockwise tilt of a large square frame tends to cause a misperception of the orientation of an enclosed line (the rod), with the rod appearing to tilt in the direction opposite the frame (Figure 1A). In the STI, the perceived orientation of a center grating is biased away from the orientation of a grating contained in a surrounding annulus (Figure 1B). Dyde and Milner (2002) have previously demonstrated that sensorimotor responses (i.e., reaching to “mail” a plastic card through the bars of the center grating in the STI, or grasping the rod in the RFI) are differentially sensitive to these illusions, with movements significantly affected by the STI but showing no effects of the RFI. The authors interpreted this pattern of results according to the Two Visual Systems model, suggesting that the contextual effect of the surround grating in the STI must occur early enough in visual processing (e.g., primary visual cortex) that the effects subsequently filter into both the ventral and dorsal streams, whereas the mechanism driving the RFI must occur deep within the ventral stream of visual processing (therefore leaving the dorsal, action stream immune to the illusion).

Although Dyde and Milner (2002) used the Two Visual Systems model to account for the differential effects of the STI and RFI on sensorimotor responses, the pattern of results can also be fully explained by the Two-Wrongs model, if the RFI, but not the STI, is associated with a distortion in the egocentric reference frame that would set the stage for a cancellation of errors. Indeed, the STI is thought to be a perceptual repulsion effect caused by the mutual inhibition of the populations of neurons encoding the orientations of the center and surround gratings (Blakemore et al., 1970; Carpenter and Blakemore, 1973; Poom, 2000; Song et al., 2013) and, as such, should have no impact on the egocentric reference frame. Since target encoding would be in error due to the orientation contrast effects, but sensorimotor responses would be guided within a veridical reference frame, there would be no cancellation of errors, and thus the illusion should be reflected in the endpoint of the movement. The RFI, in contrast, is thought to be driven by two separate mechanisms, depending on frame size, with small-frame versions of the illusion thought to be caused by the same type of perceptual repulsion effect associated with the STI (Goodenough et al., 1979; Cian et al., 1995; Spinelli et al., 1995), but with large-frame versions driven by a visuovestibular distortion of the egocentric reference frame manifest as a bias in the orientation of perceived vertical (Ebenholtz and Benzschawel, 1977; Sigman et al., 1979). Although the RFI at any given frame size is thought to be driven by a weighted summation of both the orientation contrast and visuovestibular effects, the visuovestibular distortions typically outweigh the orientation contrast effects when the size of the frame has a visual angle of 10° or more (Gogel and Newton, 1975; Zoccolotti et al., 1993). Thus, if the observer is asked to reach for the rod in a large-frame version of the RFI as was done in the study of Dyde and Milner (2002), where the frame covered a visual angle of at least 54°, the orientation of the rod will be incorrectly encoded within the distorted reference frame (causing the perceptual illusion), but the movement will be driven within the same distorted reference frame such that the errors will cancel and the movement will be accurate.

The Two-Wrongs model predicts that a specific class of illusions—those that are caused by a distortion of the observer's egocentric reference frame—will have differential effects on perception and action due to a cancellation of the errors of target encoding and motor guidance within the distorted reference frame, rather than to a clear separation of associated visual processing streams as is suggested by the Two Visual Systems model. Here, we will test the predictions of the Two-Wrongs model by comparing the possible dynamic distortion of the egocentric reference frame associated with the RFI (Experiment 1) and STI (Experiment 2), and assessing the possible effects of these distortions on perception and action, for both immediate and delayed movements.

## Experiment 1: the rod-and-frame illusion

In Experiment 1, we compared the effects of a large-frame version of the RFI on perception and action. Based on the results of previous studies, and the assumptions of the Two-Wrongs model, several predictions were formed, as follows.

1. We predict that there will be a dissociation of the effect of the illusion on perception and action, with perception prone to the illusion while actions are immune. Findings that fit this prediction will replicate the general findings of Dyde and Milner (2002) and Li et al. (2008), but further extend them by demonstrating that eye movements aimed in the direction of the rod, like reaching movements, can be accurate in spite of the perceptual illusion. This prediction of a similarity in the effects of the illusion on saccades and reaching movements is predicated on previous demonstrations of a common reference frame for the guidance of hand and eye movements (Andersen et al., 1998; Scherberger et al., 2003) and, more specifically, on previous findings of parallel egocentric biases induced by the Roelofs effect for hand and eye movements (Dassonville et al., 2004).
2. We predict that the tilted frame of the RFI stimulus will cause a distortion of the participant's egocentric reference frame, with the perception of the vertical direction biased in the direction of the frame's tilt (Ebenholtz and Benzschawel, 1977; Sigman et al., 1979). The magnitude of this distortion will be measured using a novel task in which the participant is asked to make an eye movement from a central fixation point to the topmost point of a surrounding response circle.
3. We predict that the magnitude of the distortion of the egocentric reference frame will be proportional to the magnitude of the perceptual illusion on average (replicating the findings of Li et al., 2008), but also when comparing the effects within individual participants. Findings that fit this prediction will provide strong evidence that the perceptual effects of the RFI are directly caused by the distortion of the egocentric reference frame, at least in the large-frame version of the illusion used here.
4. We predict that the distortion of the egocentric reference frame caused by the RFI will be transient (as is the distortion caused by the Roelofs-inducing frame, Dassonville and Bala, 2004), with the effect dissipating during a 4-s delay from the time that the frame is extinguished.
5. Finally, we predict that this delay-related dissipation of the reference frame distortion will be accompanied by a proportional growth of the illusion represented in delayed sensorimotor responses, since the error of motor guidance after the delay will no longer match the earlier error of target encoding, causing an inadequate cancellation of errors. Findings that fit this last prediction will, importantly, demonstrate the requirement for matched errors in target encoding and motor guidance in order to attain the circumstances that allow for the cancellation of errors described by the Two-Wrongs model of visual processing.

### Material and methods

#### Participants

Twenty participants (mean age 20.0 years, *SD* = 4.0; 75% female) were recruited from the University of Oregon Psychology Human Subjects Pool and participated in the experiment in exchange for course credit. All participants had normal or corrected-to-normal vision, and had no known neurological deficits. Participants provided informed consent in accordance with a protocol approved by the University of Oregon Institutional Review Board.

#### Apparatus

In a completely darkened room, stimuli were back-projected (Marquee 8500 projector, Electrohome, Niagara Falls, ON, CAN, with custom-fit HD145 lenses) onto a flat semi-translucent screen (Polacoat Ultra with a DA-100 diffusion coating, Da-Lite, Warsaw, IN, USA; 137 cm wide by 102 cm tall). The participant's head was stabilized by chin and forehead rests, with the eyes 90 cm from the screen. An eye tracker (Eyelink 2000, SR Research, Kanata, Ontario, CA) was used to monitor gaze during all tasks. Before each session, measurements of eye position were calibrated with a grid of 13 visual targets. If the average error in fixation was greater than 1°, the calibration procedure was repeated until a successful calibration was achieved.

#### Stimuli

**Figure 2:**
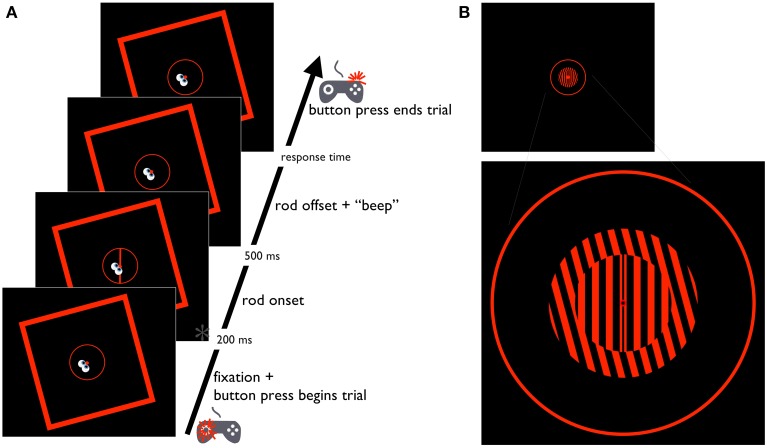
**Schematic diagram of the Perception task. (A)** Time course of the RFI Perception task. The cartoon eyes and gamepad depict the participant's gaze location and button press response, respectively. Time point marked with an asterisk (^*^) is also depicted in (**B**, upper), showing the same time point in the STI version of the task. (**B**, lower) Larger-than-scale image of the STI stimulus, providing a more detailed depiction of the fixation point, inner and outer gratings, and the response circle.

**Figure 3:**
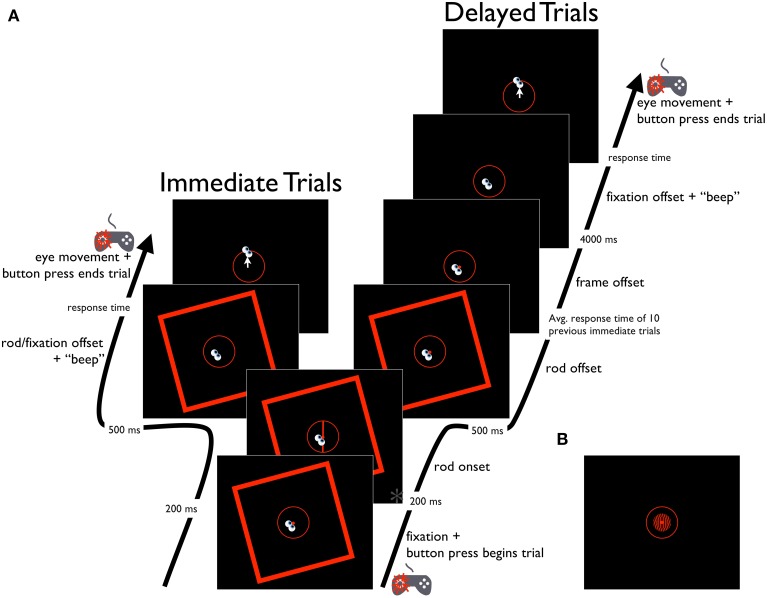
**Schematic diagram of the Saccade-to-rod task. (A)** Time course of the RFI Saccade-to-rod task, for immediate and delayed response trials. Time point marked with an asterisk (^*^) is also depicted in **(B)**, showing the same time point in the STI version of the task.

**Figure 4:**
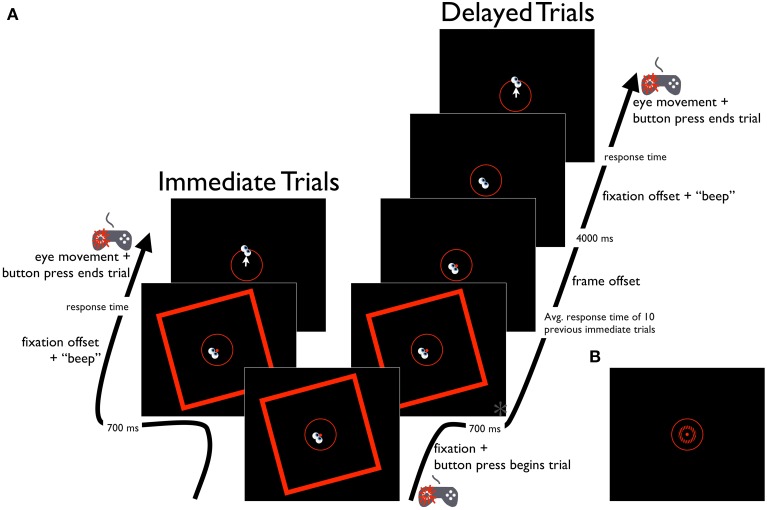
**Schematic diagram of the Saccade-to-vertical task. (A)** Time course of the RFI Saccade-to-vertical task, for immediate and delayed response trials. Time point marked with an asterisk (^*^) is also depicted in **(B)**, showing the same time point in the STI version of the task.

Stimuli were red on a black background, and included a circular fixation point (0.7° of visual angle) positioned at eye-level directly in front of the participant (Figures 2A, 3A, 4A). The fixation point was surrounded by a circle (i.e., the response circle) measuring 13.6° in diameter with a stroke width of 0.3°. Although the response circle was necessary to measure the sensorimotor responses required in two of the three tasks performed by participants (the Saccade-to-rod and Saccade-to-vertical tasks; see below), it was also included in the other task (the Perception task) for consistency—given its circular shape, it was assumed that its inclusion would have no impact on the magnitude of the illusory effects in the three tasks. The fixation point and response circle were surrounded by a square frame (38.8° of visual angle on a side, and a stroke width of 0.8°) that was tilted 15° clockwise or counterclockwise. For two of the tasks (the Perception and Saccade-to-rod tasks), stimuli also included a tilted central line (the rod, with a length of 13.6°, and a stroke width of 0.5°) centered on the fixation point (the array of possible tilts was specific to the particular task; see below).

#### Procedure

Each participant completed three separate tasks within a single experimental session: the Perception task (a cognitive measure of the magnitude of the rod-and-frame illusion), the Saccade-to-rod task (a sensorimotor report of the rod's orientation), and the Saccade-to-vertical task (a sensorimotor measure of the distortion of the participant's spatial reference frame). Prior to each task, participants completed a practice session with trials identical to the eventual experimental trials, except that the tilted frame was excluded from the visual stimuli so that participants could gain experience in the task without being exposed to the illusion itself. After the practice trials, the participants were told that the task would remain the same in subsequent trials, but that it would be performed in the presence of a tilted frame that would make the task more difficult, and therefore should be ignored if at all possible. The order of the tasks was counterbalanced across participants.

##### Perception task

In the Perception task, participants were instructed to make a categorical judgment of the rod's orientation (i.e., tilted clockwise or counterclockwise). Each trial began with the presentation of the fixation point, response circle and tilted frame (Figure 2A). Participants were instructed to direct their gaze to the fixation point, and to maintain this gaze location throughout the trial (trials were aborted if blinks occurred, or if fixation deviated more than 1° from the location of the fixation point, with these aborted trials rerun later in the experiment). After fixation was achieved, the participant was free to initiate subsequent events in the trial with a button press (left thumb) on a gamepad controller. After 200 ms, a rod (tilted either −4°, −2°, −1°, 0°, 1°, 2°, 4° from vertical) appeared on screen, lasting 500 ms before it was extinguished with an accompanying audible tone (a more narrow range of possible rod orientations was used in this Perception task than in the Saccade-to-rod task, described below, since more extreme tilts provide little information for the characterization of the psychometric functions used to assess the perceptual effects of the illusion). Participants then made an immediate button-press response to indicate the perceived orientation of the rod, with a press of the left index finger indicating a counterclockwise tilt and the right index finger indicating a clockwise tilt. Prior to the experiment, participants performed a short block of 6 practice trials without the frame present (the practice block was repeated for one participant to ensure an understanding of the task). Participants then completed 140 experimental trials, with 10 repetitions for each combination of frame tilt (−15° or 15°) and rod orientation (−4°, −2°, −1°, 0°, 1°, 2°, or 4°) presented in random order. For each frame tilt, performance was assessed by fitting a psychometric curve to the proportion of “clockwise” responses for each rod tilt in order to derive the point of subjective equality (PSE, the degree of rotation of the rod at which responses were equally likely to be perceived as tilting clockwise or counterclockwise). The overall magnitude of the illusion was quantified by subtracting the PSE for counterclockwise-tilted frames from that for clockwise-tilted frames, then halving this value to get a measure of the effect of a single frame (negative values indicated errors in the perceived orientation of the rod that deviated in the direction opposite the frame tilt).

##### Saccade-to-rod task

The Saccade-to-rod task was used to assess the effects of the RFI on sensorimotor responses, with participants instructed to make an eye movement from the fixation point to the upper end of the rod (i.e., where the rod intersected the response circle). Trials could be one of two types (namely, immediate and delayed response types), which were randomly interspersed throughout the duration of the task. Both trial types began with the presentation of the central fixation point, the response circle, and the tilted frame. Participants then guided the eyes to the fixation point and initiated subsequent events in the trial with a button press (left thumb) on the gamepad controller (Figure 3A). After 200 ms, the rod (tilted either −5°, −3°, −1°, 1°, 3°, 5° from vertical) appeared on screen for a duration of 500 ms. In immediate response trials, the disappearance of the rod was accompanied by a simultaneous disappearance of the fixation point (concurrent with the onset of a short tone), which cued participants to make an immediate eye movement to the upper end of the rod (note that although the continued presence of the response circle provided the participant with information about the appropriate *amplitude* of the eye movement, it provided no information as to the appropriate *direction* of the movement; thus, the disappearance of the rod before movement onset meant that the direction of the eye movement could only be controlled in an open-loop manner). When the participant was satisfied that the eyes were pointing at the former location of the rod's upper end, a button press (left thumb) on the gamepad controller triggered the computer to record the eye position, extinguish the frame and response circle, and terminate the trial. In these immediate response trials, the frame was presented throughout the trial period to ensure that the entirety of the response was made under the full influence of the frame (as was the case in the Perception task), since it is known that context-induced distortions of the egocentric reference frame can diminish after an inducing stimulus is extinguished (see, for example, Dassonville and Bala, 2004). In delayed response trials, the disappearance of the rod after its 500 ms duration was followed by two delay periods. The first of these was meant to equate the amount of time that the tilted frame was visible in the immediate and delayed trial types. Since the frame was present throughout the response time in the immediate trials, the delayed trials incorporated a similar period of time in which the frame was visible after the rod's disappearance, with a duration equal to the mean response time of the previous 10 immediate trials (measured from the disappearance of the fixation point until the button press that ended the trial, with a mean of 1274 ms across all participants). After this delay, the frame was extinguished, but the fixation point remained on screen for an additional 4 s, with its eventual disappearance (and concurrent audible tone) cuing participants to make an eye movement to the remembered location of the upper end of the rod. When the participant was satisfied that the eyes were correctly aimed, a button press (left thumb) on the gamepad controller triggered the computer to record the eye position, extinguish the response circle and terminate the trial. Prior to the experiment, participants performed a short block of 12 practice trials without the frame present. Participants then completed 120 experimental trials, with five repetitions for each combination of frame tilt (−15° or 15°), rod tilt (−5°, −3°, −1°, 1°, 3°, or 5°), and trial type (immediate or delayed), presented in a randomized order. Trials were aborted if participants blinked or looked away from the fixation window before the offset of the fixation point, but aborted trials were rerun later in the experiment. Performance on each trial was assessed as the difference between the true orientation of the rod and the angle of rotation of a vector plotted from the fixation point to the final eye position on the response circle. The magnitude of the errors were averaged across trials for each of the frame orientations, and the effect of the frames was quantified by subtracting the mean errors for the clockwise-tilted frames from those of the counterclockwise-tilted frames, then halving this value to get a measure of the effect of a single frame (negative values indicated eye movements that deviated in the direction opposite the frame tilt).

##### Saccade-to-vertical task

The Saccade-to-vertical task was used to assess any distorting effects of the tilted frame on the participant's egocentric reference frame. Participants were instructed to make an eye movement from the fixation point to the topmost point of the response circle, with the expectation that a distortion in the participant's perception of the vertical direction would cause a bias in the direction of the eye movement to indicate the response circle's topmost point. The stimulus parameters in this task were similar to those of the Perception and Saccade-to-rod tasks, except that no rod was presented (Figure 4A). Each trial began with the presentation of the central fixation point, the response circle, and the tilted frame. Participants then guided the eyes to the fixation point and initiated subsequent events in the trial with a button press (left thumb) on the gamepad controller. In immediate response trials, the fixation point was extinguished 700 ms after the button press, cuing the participant to make an eye movement to the topmost point of the response circle. When the participant was satisfied that the eyes were pointing at the top of the response circle, a second button press (left thumb) on the gamepad controller triggered the computer to record the eye position, extinguish the frame and response circle, and terminate the trial (the frame was presented throughout the trial period in the immediate trials to ensure that the response in these trials was made under the full influence of the frame, as was the case in the Perception and Saccade-to-rod tasks). In delayed response trials, the tilted frame was extinguished after a period equal to 700 ms plus the mean duration of the response times in the previous 10 immediate response trials (to equate the total frame duration across the immediate and delayed trials, with a mean of 1304 ms across all participants). After the frame's disappearance, the fixation point remained on screen for an additional 4 s before its offset (and concurrent audible tone) cued participants to move the eyes to the topmost point of the response circle. When the participant was satisfied that the eyes were correctly aimed, a button press (left thumb) on the gamepad controller triggered the computer to record the eye position, extinguish the response circle and terminate the trial. Prior to the experiment, participants performed a short block of 6 practice trials without the frame present. Participants then completed 40 experimental trials, with 10 repetitions for each combination of frame tilt (−15° or 15°) and trial type (immediate or delayed), presented in a randomized order. Trials were aborted if participants blinked or looked away from the fixation window before the offset of the fixation point, but aborted trials were rerun later in the experiment. Performance on each trial was assessed as the difference between true vertical and the angle of rotation of a vector plotted from the fixation point to the final eye position on the response circle. The magnitude of the errors were averaged across trials for each of the frame orientations, and the effect of the frames was quantified by subtracting the mean errors for the clockwise-tilted frames from those of the counterclockwise-tilted frames, then halving this value to get a measure of the effect of a single frame (negative values indicated eye movements that deviated in the direction opposite the frame tilt).

### Results and discussion

Table 1 provides a summary of the response times for the different RFI tasks.

**Table 1:** **Response times (as measured from fixation point offset) for the different tasks in Experiments 1 and 2 (mean ± SD, ms)**.

**Task**	**RFI (Experiment 1)**	**STI (Experiment 2)**
Perception	614 ± 187	572 ± 159
Saccade-to-rod, immediate	1245 ± 370	1208 ± 349
Saccade-to-rod, delayed	1187 ± 327	1050 ± 343
Saccade-to-vertical, immediate	1259 ± 380	1218 ± 336
Saccade-to-vertical, delayed	1184 ± 317	1036 ± 343

**Figure 5:**
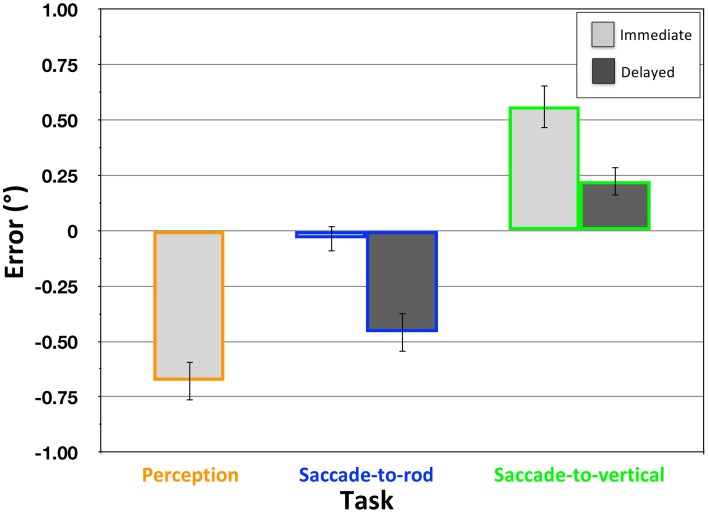
**Effect of the RFI in the Perception, Saccade-to-rod and Saccade-to-vertical tasks in Experiment 1**. Magnitude of the effect (y-axis) depicts the mean response error (°) ± 1 *SEM*, with negative values indicating errors in the direction opposite the frame tilt. Light bars represent data from the immediate response trials, dark bars from the delayed response trials; for continuity, color coding for the tasks is consistent across Figures 5–**7**.

In the Perception task, participants were asked to make a categorical judgment of the orientation of the rod (rotated clockwise or counterclockwise from vertical) within the RFI stimulus. In spite of the use of an atypical RFI stimulus (which included a response circle that was extraneous in the Perception task), the tilted frame did cause a significant bias in the perceived orientation of the enclosed rod [Figure 5; mean error = −0.68°, *SD* = 0.41°; *t*_(19)_ = −7.45, *p* < 0.001]. The negative value of the effect indicated that the perceived orientation of the rod was biased in the direction opposite the frame's tilt (i.e., a clockwise tilt of the frame caused a counterclockwise bias in the perceived orientation of the rod, and vice-versa). In contrast, when asked to move the eyes from the fixation point to the upper end of the rod in the immediate version of the Saccade-to-rod task, participants were unaffected by the frame [Figure 5; mean error = −0.03°, *SD* = 0.27°; *t*_(19)_ = −0.58, *p* = 0.57], with significantly smaller errors than in the Perception task [*t*_(19)_ = −6.72, *p* < 0.001]. These findings demonstrate that open-loop eye movements, like reaching movements (Dyde and Milner, 2002; Li et al., 2008), show no effect of the RFI, at least when they are made immediately after the rod is extinguished.

The Saccade-to-vertical task was designed to provide a measure of the distortion of the egocentric reference frame caused by the tilted frame in the RFI stimulus, with the expectation that an illusion-related bias in perceived vertical would cause inaccurate reporting of the topmost point of the response circle. Indeed, immediate eye movements to the top of the response circle were significantly affected by the tilted frame [Figure 5; mean error = 0.56°, *SD* = 0.45°; *t*_(19)_ = 5.65, *p* < 0.001], with the mean vector of the movements biased in the same direction as the frame's tilt (e.g., a clockwise tilt of the frame caused a clockwise bias in the eye movements aimed toward the top of the response circle). The direction of this distortion in the egocentric reference frame was appropriate to account for the perceptual illusion of rod orientation measured in the Perception task. For example, if a counterclockwise-tilted frame causes the egocentric reference frame (and the perception of vertical) to be biased in a counterclockwise direction (as seen in the Saccade-to-vertical task), the use of that distorted reference frame to encode the orientation of an enclosed rod would make the rod appear to be tilted in the opposite, clockwise direction (as seen in the Perception task). However, to convincingly conclude that the distortion of the egocentric reference frame is directly related to the perceptual illusion, it is also important to demonstrate that the magnitude of the two effects are equal (albeit opposite in direction). Indeed, after reversing the sign of the data from the Saccade-to-vertical task, there was no significant difference between the mean biases reflected in the Perception task and the immediate version of the Saccade-to-vertical tasks [*t*_(19)_ = −1.25; *p* = 0.23]. Even more importantly, when comparing within participants, there was a significant correlation between the distortion in perceived vertical and the perceptual effect of the illusion [*r*_(18)_ = − 0.519, *p* < 0.05], with participants showing larger biases in the Saccade-to-vertical task also showing larger illusory effects in the Perception task. These comparisons provide strong confirmatory evidence that the perceptual effects of the RFI are driven by a distortion of the participant's egocentric reference frame (Ebenholtz and Benzschawel, 1977; Sigman et al., 1979), at least for the large-frame version of the illusion employed here.

This indication that the RFI is associated with a distortion in the participant's egocentric reference frame, suggests, in turn, that the circumstances that would allow for a cancellation of the errors of perceptual encoding and motor guidance are in place. Given this, the Two-Wrongs model of visual processing would provide a viable explanation of the accurate sensorimotor responses seen in the immediate trials of the Saccade-to-rod task (as also suggested by Li et al., 2008). However, this assertion would be further strengthened if it can be demonstrated that accurate movements *only* occur when they are performed during the period in which the reference frame is distorted (that is, when the errors of perceptual encoding and motor guidance are well matched). The delayed response trials in the Saccade-to-rod and Saccade-to-vertical tasks were designed to allow the reference frame distortion to dissipate before the onset of the sensorimotor response, with the assumption that perceived vertical would drift back toward veridical under the influence of vestibular (Brandt et al., 1994; Baier et al., 2012), somatosensory (Anastasopoulos et al., 1999) and proprioceptive cues (Bottini et al., 2001) as the influence of the tilted frame waned during the 4 s delay after the frame was extinguished (see also Dassonville and Bala, 2004). The delayed response trials in the Saccade-to-vertical task provided a direct test of this assumption. Indeed, the magnitude of the distortion in perceived vertical was significantly attenuated for this delayed condition when compared to the errors in the immediate condition [*t*_(19)_ = 3.27, *p* < 0.01]. In spite of this attenuation, though, the tilted frame still had a small effect on the delayed movements to the top of the response circle [Figure 5; mean error = 0.22°, *SD* = 0.29°; *t*_(19)_ = 3.45, *p* < 0.01], indicating either that more than 4 s is required for perceived vertical to drift back toward veridical once the frame has been extinguished, or that there is a hysteresis in the drift back toward veridical (Dassonville and Bala, 2004, demonstrated a similar incomplete drift during a 4 s period of complete darkness after the removal of a Roelofs-inducing frame).

The reduction in the distortion of the reference frame seen during the delay period of the Saccade-to-vertical task, and the logic of the Two-Wrongs model, allow us to predict that the delayed response trials in the Saccade-to-rod task will show a significant effect of the tilted frame (unlike the lack of effect seen with the immediate response trials). This is due to the fact that the error of perceptual encoding that occurred during rod presentation will be, after the delay, only partially canceled by the error of motor guidance within the (now more veridical) egocentric reference frame. Indeed, there was a significant effect of the frame in the delayed responses of the Saccade-to-rod trials [Figure 5; mean error = −0.46°, *SD* = 0.40°; *t*_(19)_ = −5.15, *p* < 0.001], with the negative sign indicating that the eye movements to the top of the rod were biased in the direction opposite that of the frame's tilt [the errors of the delayed trials also differed significantly from those of the immediate trials in the same task, *t*_(19)_ = 4.91, *p* < 0.001]. Even more specifically, the Two-Wrongs model predicts that the increase in the errors in the delayed response trials of the Saccade-to-rod task (when compared to the errors of the immediate trials) will be equal in magnitude to the decrease in the distortion of the reference frame during the delay in the Saccade-to-vertical task. Indeed, we found no significant difference in the delay-related change of the Saccade-to-rod [mean shift = − 0.42°, *SD* = 0.38°] and Saccade-to-vertical tasks [mean shift = − 0.34°, *SD* = 0.46°; *t*_(19)_ = − 0.61, *p* = 0.55].

In total, the results presented here match very well the five predictions of the Two-Wrongs model: (1) Eye movements made immediately to the top of the rod were immune to the effects of the RFI, in spite of the illusion's perceptual effect, extending the findings of previous studies that have shown a similar immunity with reaching movements (Dyde and Milner, 2002; Li et al., 2008). (2) The tilted frame caused a significant distortion of the egocentric reference frame, resulting in a bias in the participants' efforts to move the eyes to the topmost point on the response circle. (3) The magnitude of the perceptual illusion was proportional to the magnitude of the distortion of the egocentric reference frame, both on average (see also Li et al., 2008) and when comparing effects within individual participants. This finding strongly supports the hypothesis that the perceptual effect of the RFI is directly caused by the distortion of the egocentric reference frame. (4) The distortion of the egocentric reference frame was found to be transient, dissipating over several seconds after the frame was removed from the field of view, similar to the transient distortion previously shown to be caused by the Roelofs effect (Dassonville and Bala, 2004). (5) This dissipation of the egocentric distortion during the delay was accompanied by a proportional growth of the effects of the tilted frame on memory-guided movements to the top of the rod, since (as per the Two-Wrongs model) the error of motor guidance after the delay would no longer match (and would therefore inadequately cancel) the error of perceptual encoding.

**Figure 6:**
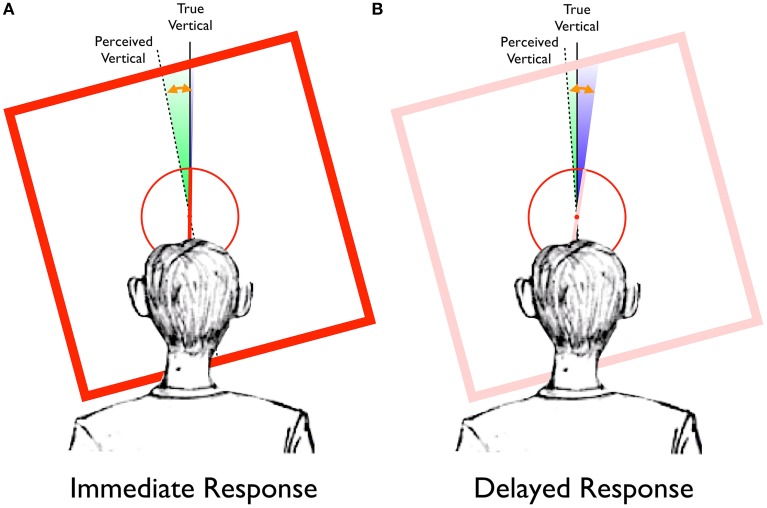
**Representation of the RFI data from Experiment 1, plotted in the context of the Two-Wrongs model. (A)** For immediate response trials, the frame-induced distortion of the egocentric reference frame (green wedge) causes perceived vertical (dotted black line) to be biased in the same direction as the frame tilt, away from true vertical (solid black line). In contrast, there is very little effect of the frame in the Saccade-to-rod task (blue wedge, barely visible to the right of true vertical). The magnitude of the perceptual effect (orange arrow) can be completely accounted for by the distortion of perceived vertical. (All effects, as depicted by the green and blue wedges and orange arrow, have been magnified by a factor of 20 for visual clarity). **(B)** For delayed response trials, the disappearance of the frame causes the distortion of the reference frame (green wedge) to dissipate, resulting in a clockwise rotation of perceived vertical toward true vertical. The remembered orientation of the rod (originally encoded with respect to perceived vertical) will be pushed clockwise as perceived vertical rotates toward true vertical, such that delayed saccades to the upper end of the remembered rod will be biased in the direction opposite the frame's tilt (blue wedge). For these delayed responses, the combined errors of the Saccade-to-vertical and Saccade-to-rod tasks (green plus blue wedges) equal the total magnitude of the perceptual illusion (orange arrow).

Figure 6 provides a graphical representation of the results of Experiment 1, in the context of the Two-Wrongs hypothesis. For immediate responses (Figure 6A) in the Saccade-to-vertical task, the reported location of the topmost point of the response circle was seen to be rotated in the direction of the frame's tilt (green wedge, whose width is proportional to the actual mean error reported above and depicted in Figure 5, but which has been magnified by a factor of 20 for visual clarity), reflecting a distortion of the typical participant's perception of vertical (dashed line). When a participant compares the orientation of the rod (truly vertical, in the example of Figure 6) to this distorted perception of vertical, its perceived orientation would be offset in the direction opposite the distortion (since the counterclockwise-tilted frame in this example would cause perceived vertical to be rotated counterclockwise by an average of 0.56°, as reported above, the upright rod would be encoded as having an orientation rotated clockwise from perceived vertical by 0.56°). Indeed, our results indicate that the magnitude of the reference frame distortion does not significantly differ from the magnitude of the perceptual illusion (depicted in Figure 6 by the length of the orange arrow, which has also been magnified by a factor of 20).

However, as discussed in the Introduction, it is thought that there are two separate mechanisms that drive the RFI depending on the size of the tilted frame, with large frames causing the illusion primarily through the visuovestibular distortion of the egocentric reference frame (Ebenholtz and Benzschawel, 1977; Sigman et al., 1979), and small frames causing it primarily through orientation contrast effects based on the mutual inhibition of neurons encoding the relative orientations of the rod and edges of the frame (Spinelli et al., 1991; Zoccolotti et al., 1993). A frame of any given size, then, would cause an illusion with a total magnitude based on a weighted proportion of the two mechanisms (Cian et al., 1995). Is it possible that our measurement of illusion magnitude is contaminated by the presence of orientation contrast effects, as well as a distortion of the egocentric reference frame? The Two-Wrongs model predicts that sensorimotor responses (such as in the Saccade-to-rod task) will only be immune to the portion of the illusion caused by the distorted reference frame. Given this, the immediate responses in the Saccade-to-rod task would be expected to reflect only the portion of the illusion caused by orientation contrast effects, if they exist. The results of Experiment 1, though, showed no significant effects of the tilted frame for these trials (depicted here as the blue wedge in Figure 6A, barely visible even after magnifying by a factor of 20), indicating that the orientation contrast effects for a tilted frame of this size are negligible. (Unpublished observations in our lab have confirmed that orientation contrast effects do cause progressively larger effects on immediate responses in the Saccade-to-rod task as the frames are diminished in size, Dassonville and Williamson, Annual Meeting of the Vision Sciences Society, 2010).

With the eye movement responses delayed by 4 s after the disappearance of the tilted frame (Figure 6B), errors in the Saccade-to-vertical task are diminished (green wedge), indicating a delay-related decrease in the magnitude of the reference frame distortion. Given that the rod was originally encoded as having a clockwise orientation with respect to the fully-distorted reference frame, the subsequent clockwise rotation of perceived vertical during the delay would tend to push the remembered representation of the rod (pink in color, in Figure 6B) with it, resulting in an increase in the magnitude of the errors for the delayed responses in the Saccade-to-rod task (blue wedge). In fact, the yoking of the decrease in the reference frame distortion with a concurrent increase in the representational error of the rod's orientation during the delay will necessarily cause the sum of the errors in the Saccade-to-vertical and Saccade-to-rod tasks (green plus blue wedges) to equal the magnitude of the original perceptual effect (orange arrow). Indeed, this is true of the current data, with no significant difference between the combined errors of the delayed responses in the two sensorimotor tasks [mean sum of errors = −0.68°, *SD* = 0.61°] and the magnitude of the original perceptual illusion [mean error = −0.68°, *SD* = 0.41°; *t*_(19)_ = 0.02, *p* < 0.98]. Thus, after the delay, the error of motor guidance within the now-somewhat-less-distorted reference frame no longer matches the original error of perceptual encoding, allowing for only an incomplete cancellation of errors. The end result is a bias in delayed sensorimotor responses, with memory-guided movements to the upper end of the rod reflecting the illusion.

While it is useful to demonstrate the robustness of the Two-Wrongs model in accounting for the dissociation of perception and action for an illusion that is caused by a distortion of the egocentric reference frame, it is equally useful to demonstrate the *specificity* of the model, by testing the predictions of the model for an illusion that is thought *not* to be driven by a distortion of perceived space. This is the purpose of Experiment 2, where we assess the relationship (or lack thereof) between the distortion of the egocentric reference frame and the simultaneous tilt illusion (STI), where an outer annulus of an oriented grating causes a center grating to appear to be rotated in the opposite direction (Figure 1B).

## Experiment 2: the simultaneous-tilt illusion

Dyde and Milner (2002) previously demonstrated that both perception and action are prone to the STI, with no significant difference between the magnitude of the perceptual illusion and the errors associated with the participants' attempts to slide a card through the middle slot of the center grating. Interpreting these results according to the Two Visual Systems model, Dyde and Milner concluded that, since the mechanism that drives the illusion is thought to have its effect in early visual processing (e.g., primary visual cortex), the distortion caused by the illusion must filter upward into both the ventral (perceptual) and dorsal (action) streams of visual processing. It is true that the illusion is thought to be brought about by mechanisms in early visual processing, with the mutual inhibition of neuronal populations encoding the gratings in the center and surround resulting in a perceptual repulsion effect (Blakemore et al., 1970; Carpenter and Blakemore, 1973; Poom, 2000; Song et al., 2013). However, interpretation of Dyde and Milner's data can be accomplished without relying on the Two Visual Systems model and its assumption of the existence of cleanly separated processing streams for perception and action. Indeed, the Two-Wrongs model fully predicts that perception and action will be equally susceptible to the STI, if the illusion is brought about by some means other than a distortion of the observer's egocentric reference frame.

In Experiment 2, we compared the effects of the STI on perception and action, using tasks with parameters identical to those of the Perception, Saccade-to-rod, and Saccade-to-vertical tasks of Experiment 1, except that the RFI stimulus in that experiment was exchanged for an STI stimulus here. Based on the results of previous studies of the STI, and the assumptions of the Two-Wrongs model, several predictions were formed, as follows.

1. We predict that perception and action will be equally prone to the STI, replicating the general findings of Dyde and Milner (2002), but also extending them by demonstrating that eye movements—aimed at the top of the center rod in the center grating of the stimulus, in a Saccade-to-rod task—will be susceptible to the illusion, just as are reaching movements.
2. We predict that the surround grating of the STI will cause no distortion of the participant's egocentric reference frame, as is expected if the illusion is solely caused by orientation contrast effects between the populations of neurons encoding the orientations of the center and surround gratings (Blakemore et al., 1970; Carpenter and Blakemore, 1973; Poom, 2000; Song et al., 2013).
3. We predict no consistent relationship when comparing, within participants, the magnitude of the perceptual illusion and accuracy in a Saccade-to-vertical task, providing further evidence that the perceptual effect of the illusion is caused by some mechanism other than a distortion of the participant's egocentric reference frame.
4. We predict that, not only will the egocentric reference frame be unaffected by the presence of the surround grating of the STI, it will remain stable during a 4-s delay after the STI stimulus is extinguished.
5. Finally, a stability in the egocentric reference frame will lead to the prediction that the magnitude of the sensorimotor effect will be unaffected by a 4-s delay in the Saccade-to-rod task, since the reference frame used to encode the center grating's orientation will be identical to that used during later motor guidance.

Findings that match these predictions will provide a useful measure of the specificity of the Two-Wrongs model for explaining why a dissociation of perception and action is expected only for those illusions (like the RFI) that are driven by a distortion in the observer's egocentric reference frame.

### Materials and methods

#### Participants

For this second experiment, a new cohort of 20 participants (mean age = 20.9, *SD* = 3.98; 50% male) was recruited from the University of Oregon Human Subjects Pool, participating in exchange for course credit. All participants had normal or corrected-to-normal vision, and had no known neurological deficits. Participants provided informed consent in accordance with a protocol approved by the University of Oregon Institutional Review Board.

#### Apparatus

The apparatus was identical to that described for Experiment 1, above.

#### Stimuli

Stimuli were red on a black background, and included a circular fixation point (0.7° of visual angle) positioned at eye-level directly in front of the participant (Figures 2B, 3B, 4B; Figure 2B, lower, presents a magnified view of the STI stimulus, for clarity). Surrounding the fixation point was a response circle measuring 13.6° in diameter with a stroke width of 0.3°. The STI stimulus contained separate round center and surround line gratings (alternating red and black bars, 1.6 cycles/°), also centered on the fixation point. The center grating had a diameter of 4.5°, with the grating capable of having an array of possible tilts specific to the task (−4°, −2°, −1°, 0°, 1°, 2°, or 4° from vertical in the Perception task, or −5°, −3°, −1°, 1°, 3°, or 5° from vertical in the Saccade-to-rod task; no center grating was presented in the Saccade-to-vertical task). The surround grating formed an annulus with an outer diameter of 7° and an inner diameter of 4.5°, with the grating tilted either −15° or 15° from vertical. Superimposed on the middle bar of the center grating was a black line (the rod) that extended the entire diameter of the center grating, with a width of 0.2°.

#### Procedure

Participants completed Perception, Saccade-to-rod and Saccade-to-vertical tasks that were similar to those of Experiment 1 (see Figures 2–4, and the Procedures of Experiment 1 for details), except that the frame of the RFI stimulus was replaced with the surround grating of the STI stimulus, and the rod of the RFI stimulus was replaced by the center grating (and the black rod in the center bar of the grating) of the STI stimulus. The timing parameters of the three tasks were also similar to those of Experiment 1, except for slight differences in the duration of the first delay period in the delayed response trials, during which the surround grating remained on the screen for a time equal to its mean duration in the previous 10 immediate response trials (with a mean of 1243 ms in the Saccade-to-rod task, and 1260 ms in the Saccade-to-vertical task, across all participants); the second delay period (after the disappearance of the surround grating but before the cue to respond) remained at 4 s. As in the Perception task of Experiment 1, participants made a categorical judgment about the orientation of the center grating (Figures 2A,B). In the Saccade-to-vertical task (Figures 4A,B), the center grating was not shown, and participants made immediate or delayed movements to the topmost point on the response circle. The shorter rod in the STI stimulus required the largest deviation in the participant's goal in the Saccade-to-rod task (Figures 3A,B), compared to that of Experiment 1. Whereas in Experiment 1 the participants made eye movements to the top of the rod in the RFI stimulus (that is, where the rod intersected the response circle), in Experiment 2 they made eye movements to “the location on the response circle that would be intersected by the rod (centered on the middle bar of the center grating) if it were extended upward along its orientation.” For each task, data analysis procedures were identical to those of Experiment 1.

### Results and discussion

Table 1 provides a summary of the response times for the different STI tasks.

**Figure 7:**
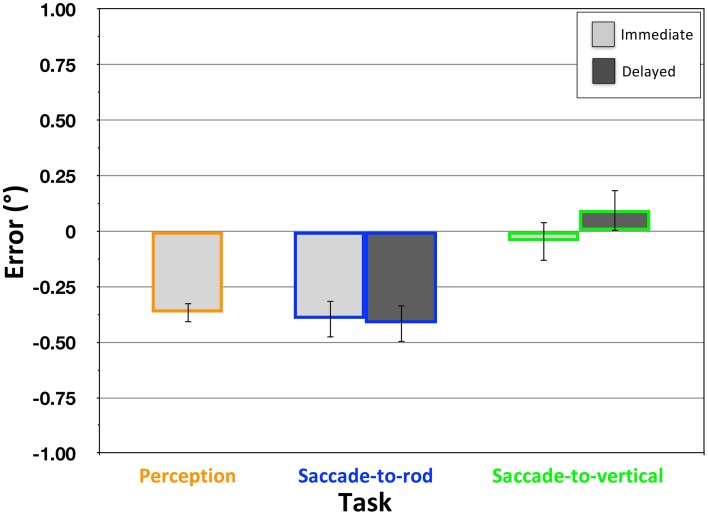
**Effect of the STI in the Perception, Saccade-to-rod and Saccade-to-vertical tasks of Experiment 2**. Magnitude of the effect (y-axis) depicts the mean response error (°) ± 1 *SEM*, with negative values indicating errors in the direction opposite the frame tilt.

In the Perception task, the participants' judgments of the center grating's orientation was found to be significantly affected by the surround grating [Figure 7; mean error = −0.37°, *SD* = 0.20°; *t*_(19)_ = −8.11, *p* < 0.001], with the center grating perceived as being rotated in a direction opposite that of the surround. In addition, when participants attempted to move the eyes from the central fixation to the location where the rod would intersect the response circle if it were extended upward (the Saccade-to-rod task), immediate movements were similarly biased by the surround grating [Figure 7; mean error = − 0.40°, *SD* = 0.38°; *t*_(19)_ = −4.72, *p* < 0.001]. A comparison of the perceptual and immediate sensorimotor effects found that they did not significantly differ [*t*_(19)_ = 0.36, *p* = 0.72], and, when comparing the effects within participants, there was a trend toward a significant correlation [*r*_(18)_ = 0.386, *p* = 0.09]. These findings replicate the results of Dyde and Milner (2002), and strongly suggest that the perceptual and sensorimotor effects of the STI have the same underlying cause.

Since the Two-Wrongs model indicates that there will be a dissociation of perception and action whenever the effects of an illusion are caused by a distortion of the egocentric reference frame, the *lack* of a dissociation with the STI leads to the hypothesis that the illusion must not be caused by such a reference frame distortion. Indeed, in the Saccade-to-vertical task, participants were able to make accurate immediate eye movements to the topmost point of the response circle, with no effect of the tilted grating in the surround array [Figure 7; mean error = −0.05°, *SD* = 0.40°; *t*_(19)_ = −.51, *p* = 0.61]. As other studies have suggested, the STI is more likely to be caused by orientation contrast effects occurring in early visual processing (Blakemore et al., 1970; Carpenter and Blakemore, 1973; Poom, 2000; Song et al., 2013), which would cause a perceptual repulsion as the orientation of the center grating is encoded within the unbiased egocentric reference frame. Given that the immediate eye movements in the Saccade-to-rod task would also be guided within this same unbiased reference frame, there would be no error in motor guidance to cancel with the error of perceptual encoding, resulting in sensorimotor responses that reflect the illusion.

With the presentation of the surround grating causing no distortion of the egocentric reference frame, one would not expect the disappearance of the surround to have any additional effect on the delayed response trials of the Saccade-to-vertical task. In fact, these delayed responses continued to be accurate, with no significant errors [Figure 7; mean error = 0.10°, *SD* = 0.43°; *t*_(19)_ = 1.04, *p* = 0.31], and no significant difference in performance between the immediate and delayed trials [*t*_(19)_ = −1.18, *p* = 0.25]. Furthermore, with a stable reference frame during the delay period, one would expect performance in the Saccade-to-rod task to also remain unchanged during the delay. Indeed, the delayed responses continued to show a significant effect of the surround grating [Figure 7; mean effect = −0.42°, *SD* = 0.44°; *t*_(19)_ = −4.23, *p* < 0.001], and there was no significant difference in the performance of the immediate and delayed responses [*t*_(19)_ = 0.28, *p* = 0.78].

The findings of Experiment 2 confirm the specificity of the Two-Wrongs model, demonstrating that accurate sensorimotor performance under illusory conditions is expected only when the illusion is one that is caused by a distortion of the observer's egocentric reference frame. In addition to the evidence presented here and elsewhere (Li et al., 2008) that the Two-Wrongs model can explain the dissociation of perception and action seen with the RFI, other studies have shown that the model can explain similar dissociations with the Roelofs effect (Dassonville and Bala, 2004; Dassonville et al., 2004) and the illusion of visually-perceived eye-level that accompanies a visible frame pitched from vertical (Li and Matin, 2005). It is likely that other illusions would also fall within this category of illusions driven by distortions of the egocentric reference frame, and would therefore also be associated with accurate movements. In contrast, illusions caused by other means (for example, contrast effects, as is the case with the STI) fail to bring about the conditions that allow for the cancellation of the errors of perceptual encoding and motor guidance.

One might argue that it could be possible to explain the difference in sensorimotor susceptibility to the STI and RFI in the Saccade-to-rod task based on a difference in the relative relationship between the central rod and the response circle in the two stimuli. Notably, in the STI version of the task in Experiment 2, the rod ended far short of the response circle, with participants required to estimate where the rod would intersect the response circle if it were extended upward, and use that estimate as the goal for the eye movement response. In contrast, in the RFI version of Experiment 1, the rod extended the entire distance to the response circle, with participants simply required to make an eye movement to the rod's upper end. Perhaps it is this difference that caused the movements in the STI version of the task to be prone to the illusion, while the accuracy of the movements in the RFI version was unaffected? To test this possibility, we performed a control experiment that replicated the immediate versions of the three RFI tasks (Supplemental Materials), but with a rod that was shorter than the diameter of the response circle, thus requiring the participants to estimate where the rod would intersect the response circle to determine the appropriate response location (as was the case in the STI tasks of Experiment 2). In this control task, the general pattern of results (large effects of the RFI in the Perception and Saccade-to-vertical tasks, small effects in the Saccade-to-rod task) were much more similar to that of Experiment 1 (compare Figure 5 and Figure S2) than to that of Experiment 2 (compare Figure 7 and Figure S2). Thus, it would appear that the difference in the results of Experiments 1 and 2 was not due to the length of the rod and whether it abutted the response circle. Instead, the difference seems attributable to the specific form of illusory context presented in the RFI and STI versions of the task.

## General discussion

The pattern of results seen with the Perception and immediate Saccade-to-rod trials in Experiments 1 and 2 very closely resembled the findings of Dyde and Milner (2002). The fact that this was true, in spite of obvious differences in stimulus and response parameters, indicates a robustness in the finding that movements (of the eyes, as tested here, or the hand, as tested by Dyde and Milner) can be accurately guided by the orientation of the rod in the context of the RFI, but inaccurately guided in the context of the STI. Thus, we can conclude that the underlying mechanisms used for guiding eye and reaching movements are affected by these illusions in a parallel or even identical fashion, as was predicted based on prior demonstrations of shared reference frames for encoding the goal locations of these movement types (Andersen et al., 1998; Scherberger et al., 2003).

However, our study went further than that of Dyde and Milner (2002), by using the Saccade-to-vertical task to directly test whether the illusions are associated with a distortion of the egocentric reference frame. Indeed, in the presence of the tilted frame associated with the RFI, saccades to the topmost point on the response circle were biased in the same direction as the frame's tilt, indicating that the context of the frame caused a distortion of the participants' sense of vertical (Figure 6A, green wedge). In turn, this distortion of perceived vertical will cause the rod of the RFI task to appear rotated in the opposite direction. As a concrete example, a counterclockwise-tilted frame that pulls perceived vertical 0.5° counterclockwise (approximating the effect size of Experiment 1 and Figure 6A) would cause a vertical rod to appear rotated 0.5° clockwise from this biased perception of vertical. The equivalent magnitudes of the sensorimotor bias of the Saccade-to-vertical task and the illusory effect measured in the Perception task, and more specifically the correlation between the individual differences in these measures, are consistent with previous suggestions that the perceptual effect of the large-frame RFI is directly attributable to a distortion of the egocentric reference frame (Ebenholtz and Benzschawel, 1977; Sigman et al., 1979). In contrast to the RFI, the tilted lines of the surround grating in the STI caused no distortion of the egocentric reference frame in the Saccade-to-vertical task, and thus we can rule out the possibility that such a distortion causes the typical perceptual illusion associated with the STI. This finding was not surprising, based on the abundance of evidence that the STI is caused by local contrast effects occurring in early visual processing (Blakemore et al., 1970; Carpenter and Blakemore, 1973; Poom, 2000; Song et al., 2013). It should be noted that Dyde and Milner (2002) also assumed that the perceptual effect of the STI is caused by orientation contrast effects in early visual processing.

Although sensorimotor responses directed to the rod of the RFI can be accurate in spite of the perceptual illusion, the Two-Wrongs model suggests that they should not be considered “immune” to the illusion. The fact that movements were affected by the illusion was most obvious when participants made eye movements to the top of the response circle in the Saccade-to-vertical task, with movements greatly affected if they occurred during the period of maximal reference frame distortion (i.e., with immediate responses), but less affected if they occurred as the distortion dissipated during the delay. The data presented here also demonstrate that even the movements to the upper end of the rod (in the Saccade-to-rod task) are prone to the illusion-induced distortion of the reference frame, but that this error of motor guidance cancels with the error of perceptual encoding if the distortion is unchanged between stimulus presentation and movement onset. Continuing the example from the previous paragraph, with a 0.5° counterclockwise rotation of perceived vertical causing a vertical rod to appear rotated 0.5° clockwise (Figure 6A), an eye movement aimed 0.5° clockwise (as is fitting, given the perceived rotation of the rod) *with respect to the counterclockwise-biased orientation of perceived vertical* would have a resulting vertical trajectory, matching the true orientation of the rod. In this way, the Two-Wrongs model suggests that although immediate sensorimotor responses *are* prone to the illusion, the cancellation of errors results in an accurate movement that masks the illusory effect. Given this view, a clear dissociation in the accuracies of perceptual and motor behaviors should not automatically imply a clear dissociation in the neural mechanisms that drive the behaviors.

Although Li and Matin (2005) and Li et al. (2008) have provided additional demonstrations that the Two-Wrongs model could be used to explain the dissociated effects of illusion on perception and action, this seemed to be true only for movements that required a fully extended arm. In the task of Li et al. (2008), participants were required to rotate their flattened hand so that its orientation matched that of the rod in a version of the RFI. When the arm was fully extended, the motor responses indicated that a cancellation of errors allowed for an accurate positioning of the hand. However, if the participant performed the task with the hand held to their side (i.e., within the midfrontal plane), this was no longer the case—participants could correctly orient the hand to vertical (indicating that the movement was made within a reference frame that was not distorted by the illusory stimulus) but showed the effects of the illusion when orienting the hand to match the rod (showing no indication of a cancellation of errors). Li et al. (2008) concluded from this that there exist two systems for guiding movements, a system for distal movements that uses a reference frame that is distorted by the illusion and another for proximal movements that uses a reference frame that is unaffected by the illusion (movements partway between these extremes would use a combination of the two reference frames in a graded fashion). With perceptual encoding of the rod always affected by the illusion, this would cause a cancellation of errors (and accurate movements) for distal movements, but no such cancellation for proximal movements. However, it is also possible to explain these results with a single reference frame used to guide both types of movements, if one accepts the possibility that the distortion of that reference frame can be non-uniform. For example, it is possible that the structure of proximal space in the reference frame is governed more by proprioceptive, somatosensory and vestibular cues and less by visual cues, while the structure of distal space is governed more by visual cues and less by proprioceptive, somatosensory and vestibular cues. In this scenario, the RFI would cause a twisting distortion of the reference frame, rather than a uniform rotation. Thus, with a fully extended arm, the error of motor guidance would fully cancel the error of perceptual encoding, but with the hand located more proximally, the error of motor guidance would only partially cancel the error of perceptual encoding. A similar non-uniform distortion of the egocentric reference frame could explain the proximal/distal dissociation seen with the illusion of visually-perceived eye-level associated with a pitched-from-vertical frame (Li and Matin, 2005), with the frame causing the vector of perceived eye-level to angle downward (or even bend downward) from the eyes, as opposed to translating downward.

Given that the visual processes that form the basis of the Two-Wrongs model allow for accurate behaviors in spite of perceptual illusions, it might be tempting to consider this to be an active, compensatory mechanism that has evolved as a way to overcome potential motor errors. However, we feel that it was more of a matter of processing efficiency, rather than error reduction, that served to shape the mechanisms described by the model. Whereas the Two Visual Systems model proposes two redundant reference frames for separately encoding an object's spatial attributes for perception and action, the Two-Wrongs model proposes simply that the brain makes use of a single reference frame (or, more likely, a single *system* of reference frames; McGuire and Sabes, 2009; Chang and Snyder, 2010; Pertzov et al., 2011) for encoding an object's spatial attributes in order to both form a perceptual representation of the object and guide movements to allow for interactions with the object (regardless of the effector, Andersen et al., 1998; Scherberger et al., 2003). A fortunate byproduct of this efficient use of a single system of reference frames for both perceptual encoding and motor guidance, as we have described, is the cancellation of illusory biases, allowing for accurate movement in spite of perceptual illusions.

While the findings reported here provide support for the Two-wrongs model in its explanation of the dissociation of the effects of the RFI on perception and action, it must be asked whether the dissociation can also be explained by the Two Visual Systems model (Milner and Goodale, 1995). Indeed, the Two Visual Systems model has already been used in an attempt to explain the lack of an effect of the illusion on reaching movements (Dyde and Milner, 2002), by suggesting that the illusion is driven by neural processes contained within the ventral stream of visual processing, leaving actions produced by the dorsal stream immune. However, a recent study from our laboratory found that the neural activity within ventral stream cortical structures was uncorrelated with illusion-related distortions of the egocentric reference frame, while the activity in a dorsal stream structure (the superior parietal lobule) did show a correlation (Walter and Dassonville, 2008). Even more strikingly, a deactivation of this same superior parietal region with transcranial magnetic stimulation was shown to directly modulate the magnitude of the RFI (Lester and Dassonville, 2014). Together, these two studies provide convincing evidence that even the *perceptual* effects of the RFI are brought about by processing in the dorsal stream, not the ventral stream, thus eliminating the whole basis by which the Two Visual Systems model was proposed to account for the perception/action dissociation associated with the RFI and other illusions caused by distortions of the egocentric reference frame.

As described here, the Two-wrongs model can explain the dissociation of perceptual and sensorimotor susceptibilities to illusions caused by distortions of the observer's egocentric reference frame, without assuming the existence of separate and distinct neural pathways for perception and action. However, one should not make the error of overextending the claims of the Two-wrongs model by assuming that it can provide an explanation for all prior and future reports of dissociations of perception and action. For example, reports of a similar dissociation for the Ebbinghaus illusion (Aglioti et al., 1995) cannot easily be explained by the Two-wrongs model, since that illusion is clearly not brought about by a distortion in the observer's egocentric reference frame. More generally, though, the Two-wrongs model, and the findings reported here, should serve as a reminder that behavioral dissociations should not automatically imply the existence of separate neural pathways for guiding the associated behaviors. Indeed, Franz and colleagues (e.g., Franz and Gegenfurtner, 2008) have examined the effects of the Ebbinghaus illusion on perceptual judgments and grasping movements, concluding, contrary to Aglioti et al. (1995), that there is a single internal size estimate that, under certain circumstances, can lead to apparent differences in the accuracies of these responses. For example, differences in the relative sensitivities of perceptual and grasping measures can cause an apparent underestimation of the illusory effect on the sensorimotor response if steps are not taken to adequately compensate for the different sensitivities (Franz, 2003). In addition, vision of the hand can increase the accuracy of closed-loop grasping movements in the Müller-Lyer illusion, but perceptual judgments seem to receive no such benefit (Franz et al., 2009; see Westwood and Goodale, 2011, and Schenk et al., 2011, for additional in-depth reviews of the evidence for and against a Two Visual Systems explanation for the Ebbinghaus, Müller-Lyer and similar illusions). Thus, it seems that there are multiple possible causes for dissociations in perception and action besides the one proposed by the Two Visual Systems model.

### Conflict of interest statement

The authors declare that the research was conducted in the absence of any commercial or financial relationships that could be construed as a potential conflict of interest.
