# Supplementary material for: The Two-Wrongs model explains perception-action dissociations for illusions driven by distortions of the egocentric reference frame
Source: Front Hum Neurosci. 2015 Mar 18;9:140. doi: 10.3389/fnhum.2015.00140 (PMC4364168; doi:10.3389/fnhum.2015.00140)
Supplement: Supplementary file 1 [file DataSheet1.PDF]

Supplementary Material

The Two-Wrongs model explains perception-action dissociations for illusions driven by distortions of the egocentric reference frame

Paul Dassonville[1]*, Scott A. Reed[1]

[1]Department of Psychology and Institute of Neuroscience, University of Oregon, Eugene, OR, USA

*** Correspondence:** Paul Dassonville, Department of Psychology, 1227 University of Oregon, Eugene, OR, 97403, USA
prd@uoregon.edu

1. Supplementary Data

Although the patterns of sensorimotor responses in the immediate trials of the Saccade-to-rod tasks with the rod-and-frame (RFI, Experiment 1) and simultaneous-tilt (STI, Experiment 2) stimuli differed in the same way as had earlier been demonstrated in the analogous tasks of Dyde and Milner (2002), one concern is whether such differences may have been observed in the current study due to differences in the stimulus characteristics of the rod across Experiments 1 and 2. Specifically, the rod in the RFI stimulus had a length equal to the diameter of the response circle, while the rod in the STI stimulus was considerably shorter, allowing for a gap between it and the response circle. Thus, in Experiment 1, participants were required simply to make a saccade to the location where the rod abutted the response circle, while in Experiment 2 participants were required to make a saccade to the "location where the rod would intersect the response circle if it were extended upward along its orientation." To ensure that the particular pattern of perceptual and sensorimotor responses in Experiment 1 were not solely due to the rod's length extending to the response circle, a control experiment was conducted in which immediate response versions of the Perception, Saccade-to-rod and Saccade-to-vertical tasks were performed with an RFI stimulus containing a shorter rod that did not abut the response circle. (The data presented here were from a single condition in a larger study that specifically tested the effect of frame size on the magnitude of the RFI; Dassonville & Williamson, Annual Meeting of the Vision Sciences Society, 2010.)

1.1. Material and Methods

1.1.1. Participants

Fifty-five additional participants (mean age 19.7 years, *SD* = 2.1; 43% female) were recruited from the University of Oregon Psychology Human Subjects Pool, participating in exchange for course credit. All participants had normal or corrected-to-normal vision, and had no known neurological deficits. Participants provided informed consent in accordance with a protocol approved by the University of Oregon Institutional Review Board.

1.1.2. Stimuli

The apparatus and stimuli were similar to those Experiment 1 in the present study (Figure S1A), with the exception of a considerable reduction in the size of the rod (to a length of 6.8° and a width of 0.2°, from 13.6° and 0.5°, respectively, in Experiment 1), and a smaller reduction in the size of the tilted frame (to 35.9°, from 38.8° in Experiment 1). The response circle was the same size (13.6° in diameter) as that of Experiment 1, resulting in a 3.4° gap between the response circle and either end of the enclosed rod (Figure S1B). Frame and rod tilts were identical to those of Experiment 1.

1.1.3. Procedure

Each participant completed the same three tasks as performed in Experiments 1 & 2, with stimulus and response timing parameters identical to those of the immediate response conditions (the delayed response conditions were not tested). As in Experiment 1, participants were asked to provide a categorical judgment of the rod's orientation (clockwise or counterclockwise tilt) in the Perception task, and make a saccade to the topmost point on the response circle in the Saccade-to-vertical task. In the Saccade-to-rod task, the shortened rod required the participants' task to change to that of Experiment 2; namely, to make a saccade to the "location where the rod would intersect the response circle if it were extended upward along its orientation."

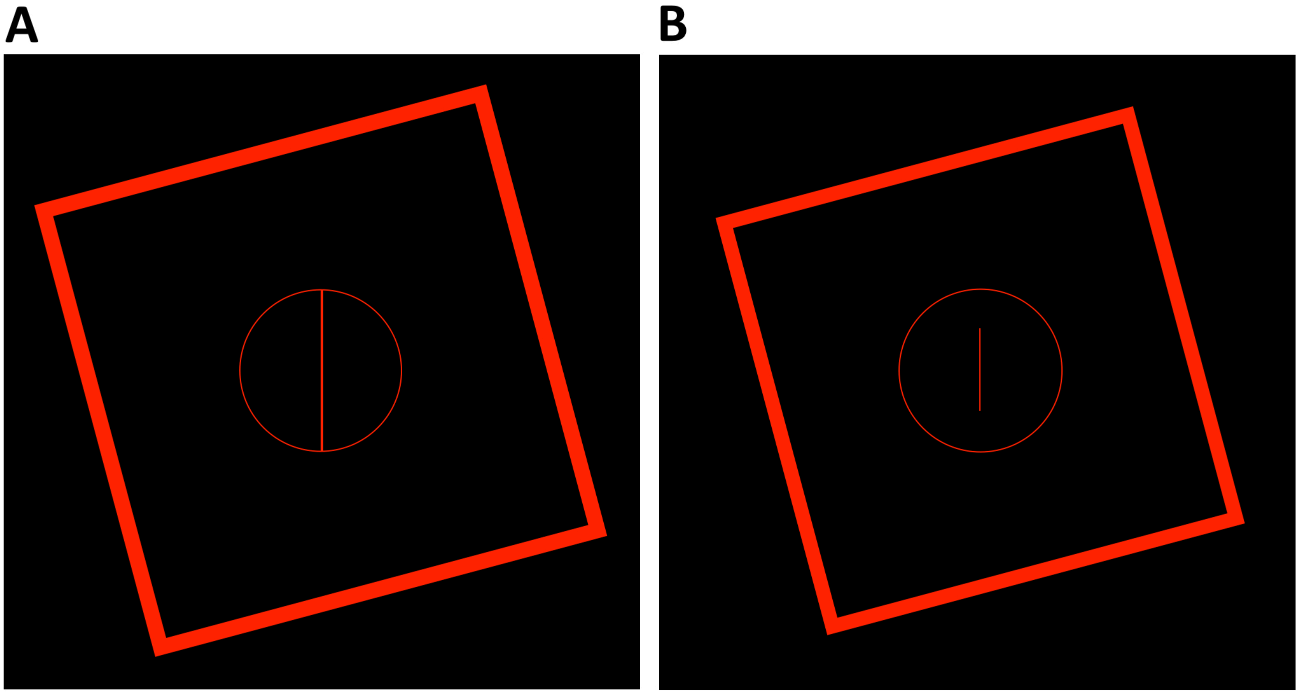

Figure S1. The RFI stimuli from Experiment 1 (A) and the control experiment (B). The size of the rod was reduced in the control experiment to include a gap between the rod and the response circle. Stimuli are shown to scale.

1.2. Results and Discussion

In the Perception task, the tilted frame was again found to cause a significant bias in the perceived

orientation of the enclosed rod (Figure S2; mean error = –1.75°, *SD* = .77°, $t(54) = -16.82$, $p < .001$). Indeed, the magnitude of this perceptual effect was significantly larger than that seen in the Perception task of Experiment 1 ($t(73) = 5.90$, $p < .0001$), where the effect had a magnitude of –.68° (note the difference in scales between Figures 5 and S2). Possible causes of this increase in the magnitude of the RFI will be addressed below.

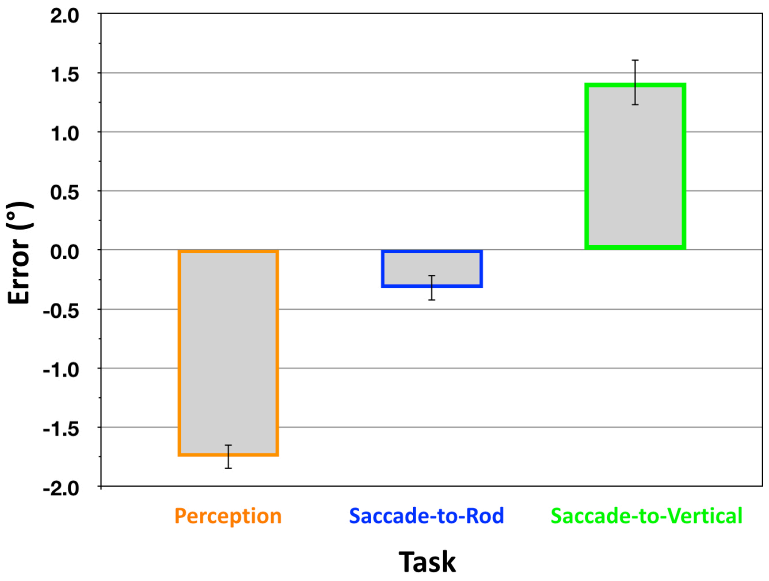

Figure S2. Effect of the RFI in the Perception, Saccade-to-rod and Saccade-to-vertical tasks in the control experiment. Magnitude of the effect (y-axis) depicts the mean response error (°) ± 1 *SEM*, with negative values indicating errors in the direction opposite the frame tilt. For continuity, color coding for the tasks is consistent with Figures in the main experiments.

In the Saccade-to-vertical task, there was again a significant distortion in the egocentric reference frame, with immediate eye movements toward the top of the response circle biased in the same direction as the frame's tilt (Figure S2; mean error = 1.42°, *SD* = 1.42°, $t(54) = 7.40$, $p < .001$; as with the Perception task, this effect was significantly larger than the .52° effect seen in Experiment 1, $t(73) = 2.65$, $p < 0.01$). Consistent with the results from Experiment 1, individual differences in the distortion of the egocentric reference frame were significantly correlated with the perceptual effect of the RFI ($r(52) = -.40$, $p < .01$), with larger illusion effects in the Perception task associated with larger biases in the Saccade-to-vertical task.

In the Saccade-to-rod task, a small but significant bias was found for immediate eye movements aimed at the location in which the rod would intersect the response circle if it were extended upward, with responses biased in the direction opposite the frame's tilt (Figure S2; mean error = –.32°, *SD* = .78°, $t(54) = -3.05$, $p < .01$). However, the magnitude of this effect did not differ from that of Experiment 1 ($t(73) = 1.62$, $p = .11$), indicating that rod length had no significant effect on the magnitude of the errors in the Saccade-to-rod task. Importantly, the magnitude of this bias in the Saccade-to-rod task was significantly smaller than that of the illusion measured in the Perception task ($t(54) = -11.35$, $p < .001$), indicating a decreased sensorimotor susceptibility to the RFI similar to that seen in Experiment 1 and the results of Dyde and Milner (2002).

A direct comparison of Figures S2 and 5 demonstrates a high degree of similarity between the

overall pattern of results seen in this control experiment and Experiment 1, with errors in the Perception task equivalent in magnitude to those of the Saccade-to-vertical task, whereas errors in the Saccade-to-rod task were significantly smaller. In contrast, a comparison of Figures S2 and 7 demonstrates that the pattern of results in the control experiment was substantially dissimilar to that in Experiment 2, where the effects of the STI were tested. In sum, these findings suggest that the overall pattern of results is not due to the length of the rod and whether it abutted the response circle in the stimulus, but was instead dependent on whether the illusory effects were driven primarily by distortions of the egocentric reference frame (as in the RFI of the control task and Experiment 1) or orientation contrast effects (as in the STI of Experiment 2).

Despite the similarities in the overall pattern of results in this control experiment and that of Experiment 1, it is relevant to question why the Saccade-to-vertical and Perception effects were larger in the control experiment. Although it might seem that the larger effects in the control task were due to the shorter length of the rod, that variable cannot provide an adequate explanation for the difference in the magnitude of the Saccade-to-vertical task, since the Saccade-to-vertical stimulus contained no rod in either Experiment 1 or the control task. More likely, the larger Saccade-to-vertical effect of the control task was due to random differences between the subject samples in the two experiments, the small differences in frame size, or a combination of both. Regardless of the cause, the larger distortion of the egocentric reference frame seen in the Saccade-to-vertical task would be expected to translate into a similarly large illusion magnitude in the Perception task, which was the case.

2. References

Dyde, R.T., and Milner, A.D. (2002). Two Illusions of perceived orientation: one fools all of the people some of the time; the other fools all of the people all of the time. *Experimental Brain Research* 144, 518–527.
